# Optimization of Alkaline Extraction of Xylan-Based Hemicelluloses from Wheat Straws: Effects of Microwave, Ultrasound, and Freeze–Thaw Cycles

**DOI:** 10.3390/polym15041038

**Published:** 2023-02-19

**Authors:** Adrian Cătălin Puițel, Gabriel Dan Suditu, Elena Niculina Drăgoi, Maricel Danu, Gabriela-Liliana Ailiesei, Cătălin Dumitrel Balan, Daniela-Lucia Chicet, Mircea Teodor Nechita

**Affiliations:** 1 “Cristofor Simionescu” Faculty of Chemical Engineering and Environmental Protection, “Gheorghe Asachi” Technical University, Bd. Prof. Dimitrie Mangeron, No. 73, 700050 Iaşi, Romania; 2 “Petru Poni” Institute of Macromolecular Chemistry, 41A Grigore Ghica Voda Alley, 700487 Iași, Romania; 3 Faculty of Materials Science and Engineering, “Gheorghe Asachi” Technical University, Bd. Prof. Dimitrie Mangeron, No. 41, 700050 Iaşi, Romania

**Keywords:** wheat straw, severity factor, response surface methodology, xylan, hemicellulose, extraction yields

## Abstract

The alkaline extraction of hemicelluloses from a mixture of three varieties of wheat straw (containing 40.1% cellulose, 20.23% xylan, and 26.2% hemicellulose) was analyzed considering the following complementary pre-treatments: freeze–thaw cycles, microwaves, and ultrasounds. The two cycles freeze–thaw approach was selected based on simplicity and energy savings for further analysis and optimization. Experiments planned with Design Expert were performed. The regression model determined through the response surface methodology based on the severity factor (defined as a function of time and temperature) and alkali concentration as variables was then used to optimize the process in a multi-objective case considering the possibility of further use for pulping. To show the properties and chemical structure of the separated hemicelluloses, several analytical methods were used: high-performance chromatography (HPLC), Fourier-transformed infrared spectroscopy (FTIR), proton nuclear magnetic resonance spectroscopy (^1^H-NMR), thermogravimetry and derivative thermogravimetry analysis (TG, DTG), and scanning electron microscopy (SEM). The verified experimental optimization result indicated the possibility of obtaining hemicelluloses material containing 3.40% glucan, 85.51% xylan, and 7.89% arabinan. The association of hot alkaline extraction with two freeze–thaw cycles allows the partial preservation of the hemicellulose polymeric structure.

## 1. Introduction

Biomass is recognized as a sustainable, renewable, and virtually limitless resource. Therefore, biomass waste-to-energy valorization technologies are required [1,2,3,4]. Agricultural biomass waste is equally appealing [2,3] as a source of energy [5] and/or basic chemicals [6]. As a result, the biomass utilization pathway is critical for maximizing profits, while covering the energy sector and chemical demand [7].

Wheat straws (WS) are a significant component of agricultural waste biomass [8], available virtually worldwide in massive amounts and with multiple ways of valorization, [8,9,10,11,12,13] such as bioethanol [9,14], biofuels [13,15,16], packaging materials [17], fertilizers [15], sugars [18,19], and others. Each WS utilization pathway, as with biomass in general, corresponds to a specific technology, and each technology is based on a particular type of pretreatment [20,21]. Some sequences may be similar for many technologies (e.g., mechanical stages, such as chopping and sieving; washing; drying; mixing); however, others are particular and correspond to the final, desired product (e.g., sol-gel and carbonization to produce wheat straw-derived magnetic carbon foams [22]). The standard mechanical and physical steps are usually followed by more aggressive physical, chemical, and biochemical pretreatments that are designed to (i) break down the covalent cross-linkages among lignin, cellulose, and hemicellulose; (ii) reduce the crystallinity of the cellulose in the cell walls of wheat straw [23,24]; and finally, (iii) separate the main components [21,25,26].

The separation of the three main constituents of lignocellulosic materials is generally recognized as the most challenging stage of the lignocellulosic biomass valorization process [27], regardless of the product sought or the technological sequence used [21,28,29]. Chemical pretreatments, such as acidic, alkaline, organic solvent, ionic liquid, ammonia, and ozone, can be used. Each extraction procedure affects the structure of the obtained material in a specific manner [21,25,29,30]. Recently, deep eutectic solvent-based methodologies attracted a lot of attention [31] for effective lignin extraction from various types of biomass, including wheat straw [32], bamboo [33], and *Triarrhena lutarioriparia* [34].

For example, if the lignocellulosic biomass is used as a feedstock for biofuel production lines, then the overall aim of such pretreatments would be a high removal rate of lignin, known for its inhibitory effect [35]. Consequently, the use of relatively harsh conditions for removing lignin is accompanied by removing hemicelluloses [36]. Alternatively, suppose the objective of the biomass processing technology includes the potential recovery of hemicelluloses and of the remaining cellulosic fibers material for the production of papermaking pulp. In that case, the pretreatment conditions must remain within a milder range to prevent excessive hemicellulose degradation and preserve a portion of them in the remaining pulp. This is due to the significance of hemicelluloses as paper-strength promoters [37,38,39].

Several benefits (efficient hemicellulose separation, effective removal of acetyl groups, mild reaction conditions, less sugar degradation, furan derivatives formation, and relatively low operation costs) [40,41] suggest that alkaline pretreatments are very effective for improving extraction yields in the case of biofuels’ production processes [11,42,43,44,45]. However, to make the process economically viable, the selectivity must be increased, the pretreatment time reduced, and the chemicals recycled [41,45]. Furthermore, by separate usage or combining the alkaline treatment with physical techniques, such as microwave [46,47,48,49], ultrasound irradiation [18,19,41,49,50,51], or freeze–thaw (FT) cycles [44,47,52], the alkaline extraction can be improved even further taking into account the final objectives.

The current study aims to optimize the extraction and separation of hemicellulose from a highly available lignocellulosic crop residue biomass category—wheat straws (*Triticum aestivum* L.). Chemical characterization of the raw materials was performed on straws from three wheat varieties, which were mixed to simulate an authentic warehouse situation. Three types of hemicellulose alkaline extraction were performed in the screening phase of the study: (i) preceded by freeze–thaw cycles; (ii) ultrasound-assisted; and (iii) microwave-assisted. The best results were obtained for two freeze–thaw cycles, followed by alkaline extraction; thus, this strategy was chosen for further modeling and optimization. The selection of the wheat straw treatment sequence is justified by the possibility of industrializing the process and integrating it into an agri-waste fractionation facility that produces both xylan-based hemicelluloses and papermaking fibers.

The wheat-straw-separated hemicellulose’s chemical structure was analyzed using high-performance chromatography (HPLC), Fourier-transformed infrared spectroscopy (FTIR), proton nuclear magnetic resonance spectroscopy (^1^H-NMR), thermogravimetry, and derivative thermogravimetry analysis (TG, DTG). Based on the results, the purity and the content in terms of components such as xylan, arabinan, and glucan were established.

Response surface methodology (RSM) was used to model and optimize the selected extraction procedure. In addition, to reduce the total number of experiments, the temperature and extraction time have been combined in a single parameter, which will be further referred to as the severity factor [53].

The novelty of the current work is sustained by: (i) screening: trial analysis to determine the most effective complementary pretreatment for hemicellulose alkaline extraction (Section 3.2), taking into account the technical and economic advantages, and disadvantages of each tested combination (Section 3.6); (ii) experimental design (Section 3.3): the experimental analysis of the selected procedure to unravel the influence of parameters on the process efficiency; and (iii) process optimization (Section 3.4): the identification of the optimal conditions that lead to a maximization of extraction considering different multi-objective criteria (simultaneous maximization of some outputs and minimization of others). To the best of the author’s knowledge, such an approach for wheat straw alkaline extraction has never been reported.

## 2. Materials and Methods

### 2.1. Analysis Methodology and Equipment

The raw materials used in this study consisted of Otilia, Sorial, and Izvor wheat straw varieties collected from Romanian farmers. For further experimental work, equal portions of the straw were mixed to avoid differences in results obtained by individual processing of straws from the various mentioned varieties. This also helps find a situation closer to the reality of the industry when different types are collected in the same warehouse. Preliminary processing of the wheat straw included grinding and sieving. Classical analytical procedures were involved in establishing the chemical composition in terms of both significant (polysaccharides and lignin) and minor constituents: ash—TAPPI T 211 om-02 [54]; hot water extractives (denoted with HWT), which were determined by the TAPPI T 207 om-88 method [55]; and organic solvent extractives T 204 cm-97 [56]. This chemical characterization was performed for each wheat straw variety and its mixture. The NREL method [57,58] for cellulose determination involves hydrolysis to glucose in two steps of sample treatment: hydrolysis with sulfuric acid at 72% and post-hydrolysis after dilution with sulfuric acid at 4%. The acetone extractives (AE) were determined according to the ISO 14453:2014 procedure.

Acid-insoluble lignin (AIL) and acid-soluble lignin (ASL) were measured using the sulfuric acid two-stages hydrolysis method specified by the NREL/TP-510-42618 method [58]. In addition, the major polysaccharide components (cellulose and hemicelluloses) were determined following an adapted procedure to that described in [49].

The employed chromatography system—Agilent Infinity 1260 II—was equipped with Phenomenex Rezex RPM-Monosaccharide Pb^+2^ (8%) 300 × 7.8 mm, heated at 65 °C. The flow rate of the mobile phase (ultrapure water) had a value of 0.6 mL/min. The injection volume was set to 5 µL. Before injection, each sample and the standard solution were filtered using 0.2 μm syringe PTFE Roth filters. Calibration curves in the concentrations range of 0.05–0.3 g/L were plotted using solutions of 99% purity glucose, xylose, and arabinose (provided by Flucka). A Jasco V550 UV–Vis spectrometer was used to record the absorbance values at 205 nm [59].

### 2.2. Screening Phase for Extraction of Hemicellulose from Wheat Straw

An initial screening set of experiments was performed to establish the effect of individual pretreatments or alkaline extraction conditions on hemicelluloses’ removal yields. This experimental stage included:

(i) trials on FT cycles. The freeze–thawing cycles were repeated up to four times to test their impact on the efficiency of the subsequent hot alkaline (HA) extraction stage. The freezing phase in the freeze–thaw cycle trials was performed by immersing 2 g of oven-dried straw in water in polypropylene sampling vessels. The samples were frozen in a conventional freezer (−22 °C) for 60 min. Then, they were removed from the fridge and left to thaw at room temperature. After defrosting, the samples were reinserted into the freezer to start a new cycle. The extraction yields were measured after a standalone FT cycle and for FT cycles followed by HA. The HA procedure is described in our previous work [40]. For the screening trials, a 5% sodium hydroxide (NaOH) solution was used as an extractive agent at a temperature of 90 °C and 40 min at a solid-to-liquid ratio of 1:30.

(ii) ultrasound treatment. The ultrasonic extraction experiments were realized using a custom-made ultra-sonication system with a generator and horn capable of supporting 50 W at 40 kHz for 10 min. In this case, the treatment was performed in an alkaline environment without supplementary heating.

(iii) extraction under microwave heating conditions. The microwave-assisted heating was performed in an alkaline solution using a 700 W commercially available home oven (Zanussi ZMC 19 MG, microwave frequency—2.45 GHz). This procedure was applied as a potential replacement for conventional heating employed in classic HA.

### 2.3. Experimental Design of Alkaline Extraction of Hemicelluloses from Freeze–Thaw Pretreated Wheat Straw

The RSM methodology with central composite design was used to model and optimize yields after the best extraction method was determined during the screening phase. The main parameters (Table 1) for modeling the extraction of hemicelluloses from the two preliminary freeze–thaw-cycles-treated wheat straw were: (i) the severity factor is defined as the combination of extraction time and the temperature, Equation (1) [53,60]; and (ii) NaOH concentration:

(1) $$SF=\log_{10}\left(\tau *e^{\frac{T-100}{14.75}}\right)$$

where *SF* represents the severity factor; *τ* is the processing time at selected temperature *T*.

The analyzed dependent variables are: the total extraction yield (TY), the homoxylan extraction yield (XY), the total hemicellulose extracted yield (HCY), the acid-insoluble lignin removal yield (YAIL), and the acid-soluble lignin removal yield (YASL). Equations (2)–(6) indicate how the output was determined based on experimental data. Stat-Ease Design-Expert (version 7) was used for the experimental design and processing of the experiments;

(2) $$\mathrm{TY}\;\left(\%\right)=\frac{m_{1}-m_{2}}{m_{1}}\times 100$$

where TY (%) represents the total extraction yield; m_1_ is the initial mass of the treated samples, oven dried; and m_2_ is the mass of the solid residue remaining after the extractive treatment, oven dried;

(3) $$\mathrm{XY}\;\left(\%\right)=\frac{m_{X1}-m_{X2}}{m_{X1}}\times 100$$

where XY (%) stands for the xylan extraction yield; m_X1_ is the absolute mass of the xylan in the raw material sample; and m_X2_ is the absolute mass of the xylan, determined after two-stage acid hydrolysis;

(4) $$\mathrm{HCY}\;\left(\%\right)=\frac{m_{\mathrm{AX}1}-m_{\mathrm{AX}2}}{m_{\mathrm{AX}1}}\times 100$$

where HCY (%) stands for the hemicellulose extraction yield; m_AX1_ is the absolute mass of the hemicellulose—arabinoxylan in the raw material sample; and m_AX2_ is the absolute mass of the hemicellulose—arabinoxylan, determined after two-stage acid hydrolysis;

(5) $$YAIL\;\left(\%\right)=\frac{m_{\mathrm{AIL}1}-m_{\mathrm{AIL}2}}{m_{\mathrm{AIL}1}}\times 100$$

where *YAIL* (%) stands for the acid-insoluble lignin extraction yield; m_AIL1_ and m_AIL2_ are the absolute mass of the acid-insoluble lignin in the raw material and treated sample;

(6) $$YASL\;\left(\%\right)=\frac{m_{\mathrm{ASL}1}-m_{\mathrm{ASL}2}}{m_{\mathrm{ASL}1}}\times 100$$

where *Y_ASL_* (%) stands for the acid-soluble lignin extraction yield; m_ASL1_ and m_ASL2_ are the absolute mass of the acid-soluble lignin in the raw and treated sample, respectively. The NREL/TP-510-42618 method was used to determine m_AIL1_, m_AIL2_, m_ASL1_, m_ASL2_ [58].

The plan consists of thirteen experiments, including five replications at the center point. The range of parameters included in the RSM analysis (Table 1) was determined based on a series of preliminary investigations and a previous study [40]. The experimental results were then utilized to generate regression models describing the interdependence between parameters and extraction yields.

After determining the regression models for each yield based on the experimental data, process optimization was performed, considering the maximization of the total extraction yield.

### 2.4. Separation and Characterization of Extracted Hemicelluloses

Separating hemicelluloses involves the preliminary lignin separation by acid precipitation at pH 5. The subsequent treatment of the supernatant with 96% ethanol leads to hemicellulose separation. The purity of the obtained hemicelluloses depends on the amount of lignin initially present in the alkaline extraction liquor, which depends on the extraction conditions. The increased extraction time, temperature, and sodium hydroxide concentration lead to higher amounts of lignin in the HA-produced liquor. At the same time, during the hemicellulose extraction process, lignin is degraded into soluble products at a pH below 5. This aspect leads to the co-precipitation of lignin with the hemicelluloses during the graded ethanol method used to separate hemicelluloses.

In the graded ethanol precipitation method, volumes of 100 mL samples of alkali extraction liquors were neutralized to pH 5.5 by using glacial acetic acid to precipitate and remove some of the lignin co-extracted. The resulting liquor was centrifugated at 3000 rpm for 15 min in a Sorvall GLC2 equipped with an HL-4 rotor (100-mL bucket). The remaining supernatant was mixed with 200 cm^3^ of analytic purity ethanol (96%) and left to stand at 4 °C for 24 h. The precipitated hemicelluloses (HC) were separated by centrifugation for 15 min and double-washed with ethanol. Furthermore, the resulting HC samples were dried at 50 °C and prepared for HPLC analysis to determine the constituent monosaccharides residues. The HPLC system and analysis conditions were described in Section 2.1. The injection volume was increased to 20 µL to accommodate the concentration of sugars in the hemicelluloses derived hydrolysate. The exact procedure for wheat straw hemicelluloses processing before HPLC analysis was described in our previous work [40].

The FTIR spectra of the wheat straw extracted and separated hemicelluloses samples were recorded using potassium bromide disks containing finely ground samples at 1% content on an Agilent Cary 630 FTIR instrument (64 scans at 4 cm^−1^ resolution and 4000–400 cm^−1^).

Samples of 30 mg of hemicelluloses dissolved in deuterated water and pipetted into NMR tubes were used for ^1^H-NMR spectroscopy analysis. The spectra were recorded on a Bruker Avance NEO 400 MHz spectrometer, operating at 400.1 MHz for 1H nuclei, with a 5 mm four nuclei direct detection z-gradient probe using standard pulse sequences, as delivered by Bruker with TopSpin 4.0.8 spectrometer control and processing software. Chemical shifts are reported in δ units (ppm) and were referenced to the sodium 3-(trimethylsilyl)-(2,2,3,3-d4)-1-propionate (TSP) internal standard. For spectra registration, 128 scans were used.

Thermogravimetric analysis of hemicelluloses samples was carried out using a Toledo TGA/SDTA 851 instrument at a heating rate of 10 °C∙min^−1^ and a nitrogen flow rate of 20 mL∙min^−1^, using a sample weight of 2–6 mg. Ceramic pans were used to heat the samples from 25 °C to 800 °C. Mettler Stare SW 9.10 TGA/DTG software was used for data processing.

The surface morphology of the samples was studied with the help of a Vega-Tescan LMH II type scanning electron microscope, using a secondary electrons (SE) detector and an 8 kV filament voltage on a high-vacuum (HV) working module. First, the analyzed samples were taken in a dry state at the end of each extraction stage. Then, without further intervention, they were mounted directly on the holders using a double-copper adhesive strip.

## 3. Results and Discussion

### 3.1. Wheat Straw Chemical Composition

The main chemical components are cellulose, hemicelluloses, and lignin. Several methods of analysis were used (Section 2.1.) to estimate the amount of various constituents of the raw materials (Table 2). The total amount of hemicelluloses (xylan and arabinan) is denoted with HC. Table 2 is designed to compare the different types of constituents for the three varieties of wheat straw and their mixture (1:1:1), and the results do not represent the total chemical composition.

There are minor differences (1% variation) in cellulose content between the different varieties, while for the hemicelluloses content, it ranged from 24.97% to 27.27%; and for acid-insoluble lignin values, from 15.8% to 20.6%.

AE were lower than 3% for Otilia and Izvor varieties, while for the Sorial variety, a value of 4% was recorded. This includes waxes, fats, resins, and sterols. The extraction was performed using acetone; thus, the current study-reported-data have smaller values than the standard ethanol–benzene mixture until recently [61,62]. The content of hot water extractives indicates the biomass’s soluble organic materials (tannins, gums, soluble non-structural sugars, starch, and coloring substances) or inorganics, such as salts or nitrogenous material [63,64]; and the current obtained values are comparable to literature data [61].

The ash content is proportional to the amount of mineral substances present, and it is higher in non-wood biomass samples than in wood biomass samples. The experimental values obtained are consistent with those reported by other authors in similar studies [20].

### 3.2. Screening Phase for Extraction of Hemicellulose from Wheat Straw

The results displayed in Table 3 offer a comparative view of the proposed extractive treatment results. FT represents the freeze–thaw pretreatment stage; the number of cycles is given by the associated number (e.g., FT 2 indicates that the sample underwent two freeze–thaw cycles). HA 40-90 is the hot alkali extraction stage, where the former represents the time, while the latter represents the temperature, in Celsius degrees. US10-30 and US20-45 indicate ultra-sonication for 10 and 20 min at 30 °C and 45 °C, respectively. MW 10-100 represents microwave-assisted heating in an alkaline environment at 10 min and 100 °C.

The data shows that the freeze–thawing cycles enhance the alkaline extraction process, while the microwave heating or ultrasound treatment acts as a standalone extraction process. Applying microwave heating or ultrasound irradiation in an alkaline solution (with the mechanisms detailed in Section 3.6.) has similar effects to classic HA. However, the yields strongly depend on the irradiation time and frequency. Both procedures reduce the treatment time; however, temperature control is difficult and specialized equipment is required, which limits its wide adaptation in various settings.

Freeze–thawing alone removes only a tiny amount of xylan and hemicelluloses, probably water-soluble fractions, together with some lignin. Regarding the number of freeze–thaw cycles, the most effective extraction occurred after two cycles. Considering the simplicity of the laboratory setup and its potential for up-scaling, the sequence consisting of two freeze–thaw cycles, followed by hot alkaline extraction (FT 2 HA) was chosen for modeling, optimization, and product characterization.

### 3.3. The Influence of Extraction Parameters

The central composite experimental design and results for FT 2 HA are displayed in Table 4. First- and second-order polynomial regression equations were used to fit the experimental data. The equations were simplified by removing some non-significant terms, while maintaining the model hierarchy. The proposed relationships between the extraction yields (total extraction yield, xylan extraction yield, total hemicelluloses removal and lignin removal, and parameters are shown in Equations (7)–(11) (all *p*-values were less than 0.05). The detailed ANOVA analysis and the statistical indicators for these models are presented in Tables S1–S5.

(7) $$\mathrm{TY}\;\left(\;\%\right)=-6.01X_{1}^{2}+0.267X_{2}^{2}+31.8X_{1}-0.676X_{2}-0.264X_{1}X_{2}+10.4;R^{2}=0.97$$

(8) $$\mathrm{XY}\;\left(\%\right)=21.4X_{1}+11.4X_{2}-2.08X_{1}X_{2}-30.5;\;R^{2}=0.95$$

(9) $$\mathrm{HCY}\;\left(\%\right)=15.9X_{1}+8.95X_{2}-1.1X_{1}X_{2}-17.5;R^{2}=0.94$$

(10) $$\mathrm{YAIL}\;\left(\%\right)=70.4-84.1X_{1}+3.11X_{2}+1.44X_{1}X_{2}+24X_{1}^{2}-0.267X_{2}^{2};R^{2}=0.97$$

(11) $$\mathrm{YASL}\;\left(\%\right)=-4.99X_{1}-0274X_{2}+2.01X_{1}X_{2}+27.2;\;R^{2}=0.89$$

**Figure 1:**
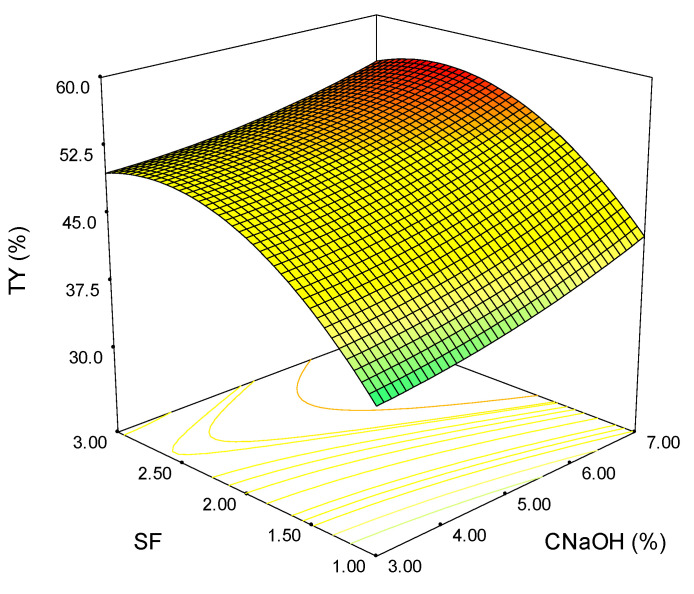
Variation of the total extraction yield as a function of severity factor and alkali concentration (the colours green-yellow-red indicate the shift from low to high values).

Figure 1 shows the tridimensional surface obtained for the total yield variation due to SF and sodium hydroxide concentration. Increasing both SF and CNaOH resulted in an increase of TY (%). At a constant value of 3% NaOH, a variation of 1 unity for SF leads to a nearly 27% rise in TY (%). The maximum value for the total extraction yield is obtained for an SF of 2.5. Over this value, TY (%) slightly decreases regardless of the alkali concentration, which can be explained by the NaOH consumption in concurrent reactions. To highlight the effect of CNaOH, the SF was held constant. At SF = 1, the increase of alkali concentration from 3 to 7 leads to a rise in TY (%) of 19.3%. The same rising trend is observed at higher SF values; however, the % is lower (12.1% for SF = 2 and 9.6% for SF = 3). Overall, SF has a higher impact on total extraction yield than CNaOH.

**Figure 2:**
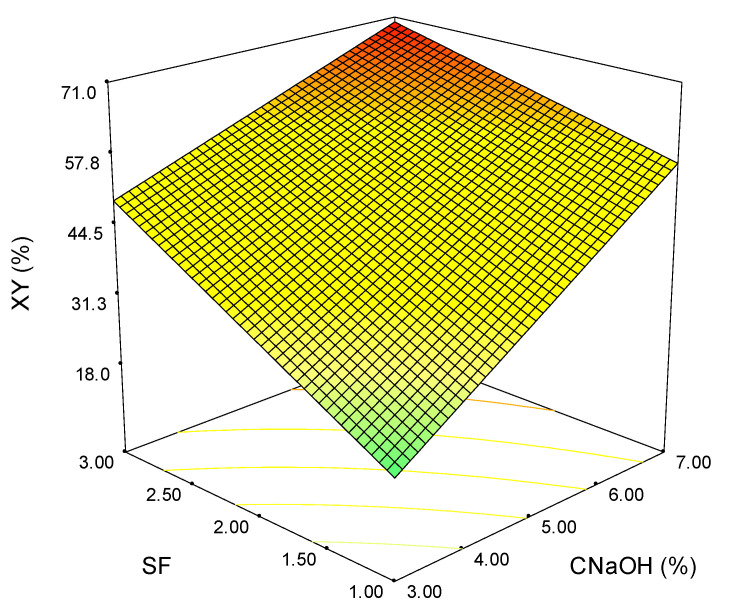
Variation of homoxylan extraction yield as a function of severity factor and alkali concentration (the colours green-yellow-red indicate the shift from low to high values).

Figure 2 shows the response surface plot obtained for the homoxylan yield variation in relation to alkali concentration and severity factor. As can be observed, the maximum XY (%) value is obtained at SF = 3 and CNaOH = 7%. Distinctively from the TY (%) where the maximum was at SF = 2.5, indifferent of alkali concentration, in this case, both parameters have a significant influence on extraction yield. Due to the considerable impact of both parameters, the homoxylan yield does not reach a plateau as for TY (%). The total effect of the two parameters has a more substantial impact on XY (%) than either of them would have on their own.

**Figure 3:**
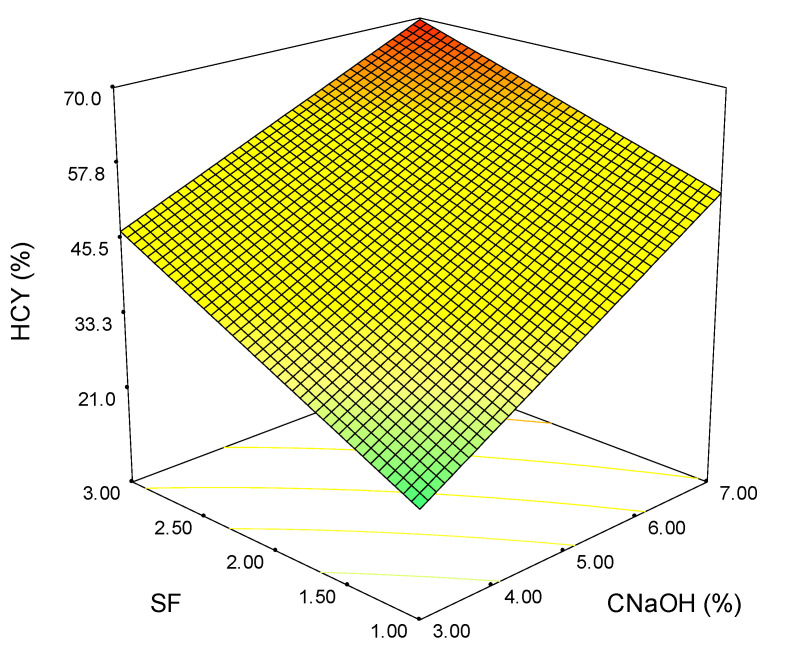
Variation of the total hemicelluloses extraction yield with severity factor and alkali concentration (the colours green-yellow-red indicate the shift from low to high values).

Figure 3 shows the response surface of the model for the total hemicelluloses extraction yield. Similar behavior as in the case of XY (%) can be observed for HCY (%); the highest extraction yield being obtained at maximum values for SF and CNaOH. However, the individual influence of the considered variables is slightly different (indicated by the slopes of the surface in relation to each parameter).

**Figure 4:**
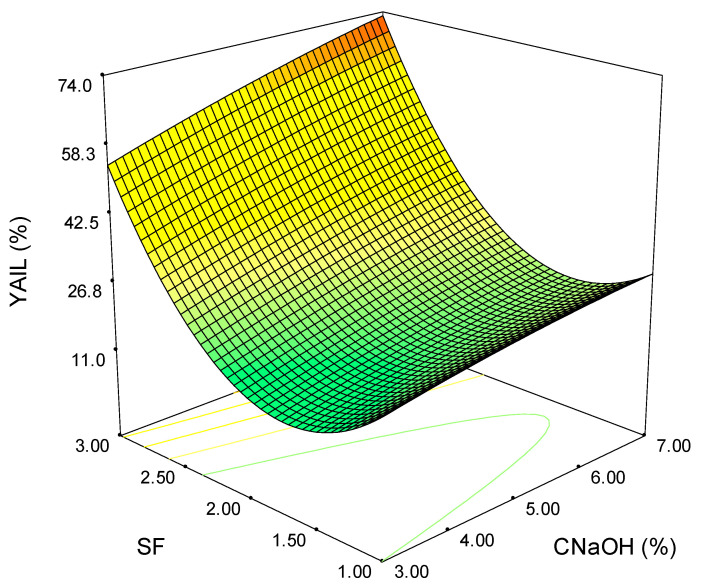
Variation of the acid-insoluble lignin removal yield on severity factor and alkali concentration (the colours green-yellow-red indicate the shift from low to high values).

Figure 4 displays the variation of YAIL (%) on the model parameters. At lower values of SF (interval 1–2), the variation has a non-linear trend; and the extraction efficiency is minimum, the increase of SF to 2 leading to a decrease in extraction yield (negative slope). On the other hand, the raise of SF to 3 leads to a rapid YAIL (%) increase (positive slope). Although the CNaOH has a low influence on the overall performance, the maximum YAIL (%) is reached at the highest alkali concentration (red-colored surface).

**Figure 5:**
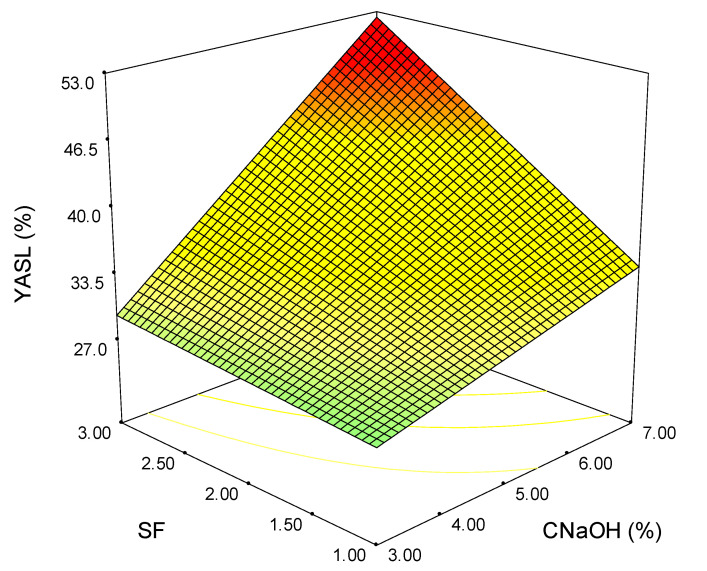
Variation of the acid-soluble lignin removal yield as a function of severity factor and alkali concentration (the colours green-yellow-red indicate the shift from low to high values).

The response surface plot for the variation of the acid-insoluble lignin removal yield is shown in Figure 5. In this case, it can be observed that the individual effects of SF and CNaOH are reduced in comparison with their combined effect. When SF = 2, YASL (%) ranges from 28.5% to 43.4% for the entire alkali concentration interval, while when CNaOH = 5, the acid-soluble lignin removal yield varies from 30.9% to 40.9% for the entire SF domain. The highest removal efficiency of the acid-soluble lignin is obtained for SF = 3 and CNaOH = 7.

Although the maximum extraction yields are obtained at the maximum values of SF and CNaOH for all the analyzed outputs, the shape of the surface plots suggests a linear dependence for homoxylan, hemicellulose, and the soluble acid lignin (Figure 2, Figure 3 and Figure 5). In contrast, for total extraction and acid-insoluble lignin (Figure 1 and Figure 4), there is a non-linear dependence. This aspect can also be observed by analyzing the determined mathematical models and the ANOVA reports (Tables S1–S5).

### 3.4. Process Optimization

After determining the models and the impact of each individual parameter was analyzed, the process was optimized considering two cases: (i) the experimental limits (this will be further referred to as O1); and (ii) extrapolation for CNaOH to 9 % (this will be further referred as O2). In both cases, multiple objectives were considered in such a manner to obtain a maximum TY (%), XY (%), HCY (%), and a minimum YAIL (%) of YASL (%). In addition, the minimization of these two outputs was considered because higher amounts of lignin in alkaline extraction liquors hinder the hemicellulose-graded ethanol separation stage. The results obtained are presented in Table 5. For O1, a severity factor of 1.63 was identified. The experimental parameters were set at 100 °C and 43 min to obtain this value. For O2, the severity factor was 1.44, corresponding to 100 °C and 25 min.

As can be observed from Table 5, in the case of extrapolation (O2), the errors between the predicted and the experimental values are higher than for O1. This is because extrapolation has a higher uncertainty and assumes the same dynamic occurs as in the experimentally verified interval, which is not necessarily true for real-world processes.

### 3.5. Product Characterization

#### 3.5.1. Component Identification

Table 6 indicates the main components of the hemicelluloses obtained through the experimental verification of the solutions provided by the optimization procedure. HC stands for hemicelluloses obtained from the wheat straw by alkaline extraction using optimized conditions and without performing the freeze–thaw cycles. The notations HC_O1 and HC_O2 were assigned for the hemicelluloses separated from alkaline extractive treatment liquor preceded by two freeze–thaw cycles for O1 and O2, respectively. As can be observed from Table 6, the freeze–thaw cycle seems to favor the xylan extraction yield versus the arabinan extraction yield.

#### 3.5.2. FTIR Analysis

**Figure 6:**
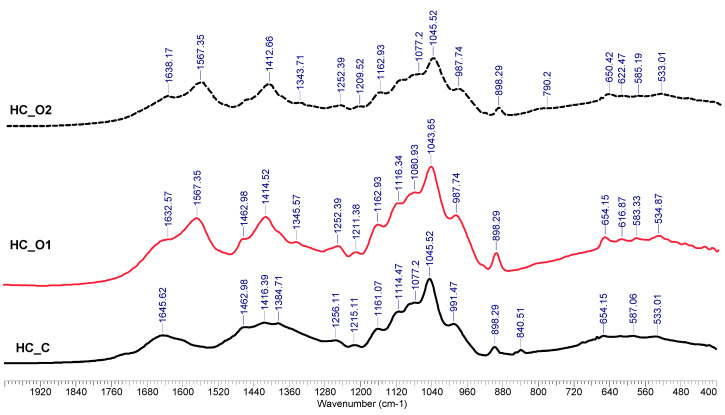
FTIR spectra of hemicelluloses separated from wheat straw by using different sequences.

The infrared spectra of wheat straw hemicellulose samples are presented in Figure 6. Figure S1 shows the complete spectra for the analyzed samples. The band occurring at ~1645 cm^−1^ was assigned to the absorbed water [64]. The absorptions at ~1550 cm^−1^ are probably caused by impurities such as co-separated lignin. Peaks shown at about 1460 cm^−1^ were assigned to the presence of the methyl groups. Peaks visible at ~1100 and ~1045 cm^−1^ result from C-O stretching in C-O-C ether linkages (the first is inter-sugar units; and the second is from intra-sugar, in the alcoholic functional group). The ~898 cm^−1^ peak is specific to β-1-4 bonds between xylose units of the xylan chain and is caused by the stretching vibration modes (both symmetric and antisymmetric) of C-O in this linkage [41].

Other bands at lower wavenumbers, such as ~690 cm^−1^, were attributed to the CH deformations—out of the plane. Peaks at ~3400 cm^−1^—stretching vibrations of O-H groups; the ~2950 cm^−1^ to -CH_2_ antisymmetric stretching, while the 2850 cm^−1^ results from -CH_2_ symmetric stretching.

#### 3.5.3. ^1^H-NMR Spectroscopy Analysis

**Figure 7:**
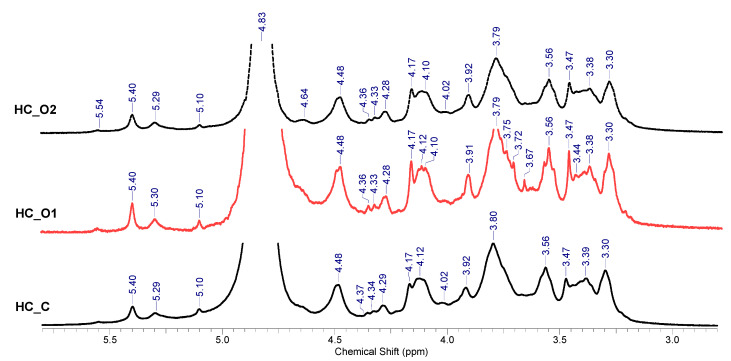
^1^H-NMR spectra of hemicelluloses separated from wheat straw.

Figure 7 shows the results obtained by ^1^H-NMR spectroscopy. The displayed spectra include three regions: (i) the region of α-anomers in the range 5.5–4.9 ppm; (ii) β-anomers’ region assigned in the interval 4.9–4.4 ppm; and (iii) the β-(1-4)-D-anhidroxylopyranose units heterocycle proton region 4.4–3.0 ppm [65]. The first region includes signals for α-L arafuranosyde residues assigned to the anomeric H1_Ara_ at 5.4 ppm and for the H4_Ara_ proton assigned at 4.3 ppm. The second region displays signals for non-substituted xylose units (4.49 ppm) and for glucuronic-acid-substituted β-xylose (4.64 ppm) [66,67,68]. For the third region, the assignment was considered as follows: H2_Ara_—4.11 ppm; H3_Ara_—3.75 ppm; H5_Ara_—3.68 ppm for the α-L arafuranosyde residues (Ara); and H5_eq X_—4.11 ppm, H4_X_—3.80 ppm, H3_X_—3.56 ppm, H5_ax_—3.38 ppm, H2_X_—3.30 ppm for the β-D-xylopyranoside residues (X).

#### 3.5.4. Thermogravimetric Analysis

As Nurazzi et al. noted in their literature review on TG analysis of cellulose fiber and respective composites, hemicelluloses have typically lower decomposition temperatures than lignin [69]. Similar conclusions were previously highlighted by other authors, which showed that the biomass component decomposition temperatures increase in the order of hemicelluloses (max. weight loss 220–315 °C) < cellulose (max. weight loss 315–400 °C) < lignin (degradation extended up to 900 °C) [70].

**Figure 8:**
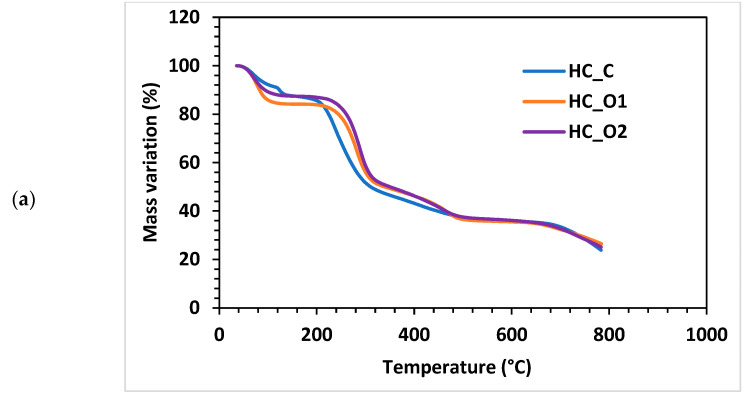

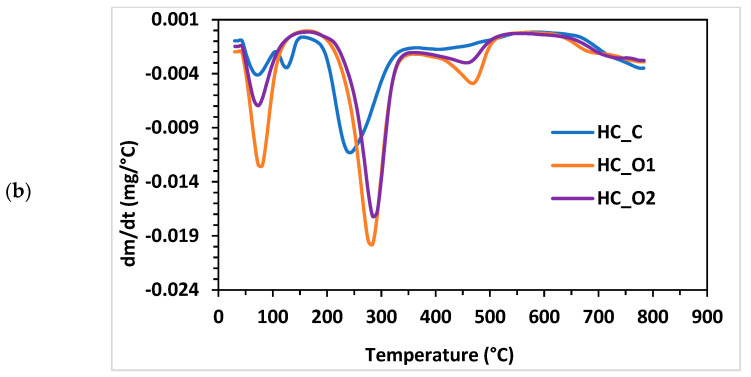
Thermogravimetric analysis: (**a**) mass-variation curves; (**b**) DTG curves.

Regarding the results obtained for the analyzed hemicelluloses, several phases are visible in the case of the mass variation graphs (Figure 8a,b). For the HC_C sample, the water removal (dehydration phase) occurs in two steps: one between 48 °C and 120 °C (mass removed yielded ~8.57%), and a short secondary step between 120 °C and 132 °C (with a DTG peak at 126 °C). Samples HC_O1 and HC_O2 displayed a single-step dehydration within the 50 °C to 101 °C range; and peaks on DTG curves occurring at 77 °C and 70 °C, respectively. The computed values of the mass loss were 15.8% for HC_O1 and 12.8% for HC_O2.

The second stage of the thermo-gravimetric test corresponds to the polymeric chain degradation phase. For the HC_C sample, this occurs between 215 °C and 295 °C, with a mass loss of ~41% and a peak at 234 °C. HC_O1 and HC_O2 showed a degradation phase occurring in similar temperature ranges: (i) 248–303 °C for HC_O1 with a DTG peak observed at 283 °C, 37.6% mass loss; and (ii) 258–309 °C for HC_O2 with a peak at 290 °C and 39.3% mass loss. The higher temperatures of corresponding HC_O1 and HC_O2 peaks during the second stage compared with HC_C indicate their better thermal stability.

In the final stage of thermal decomposition, the oxidation of the remaining char occurs. The HC_C recorded range was 360–503 °C with a peak at 414 °C (~11% mass loss). The analysis of HC_O1 and HC_O2 revealed a similar interval of 360–490 °C with peaks at 490 °C (~12% mass loss).

Peng and Wu [71] proposed a mechanism for the breakdown of the hemicellulose polymer chain that involves dehydration, fragmentation of side chains, decarboxylation and decarbonylation, and charring. Carbon dioxide, acetic acid, and pentanal are the most widely encountered degradation products; and they are all released above 250 °C in varying amounts during the various stages of decomposition [71]. In the current work, the identified peaks are in accordance with literature data [69,71], and the hemicellulose samples showed thermal stabilities comparable with xylan-based polysaccharides [40,72,73,74].

### 3.6. Mechanism of Complementary Pretreatments and Comparison with Similar Studies

#### 3.6.1. Freeze–Thaw Cycles

Freeze–thaw effects are of a mechanical nature, based on the water’s property to expand volume when frozen. Ice formation may expand, distort, and finally disrupt biomass pores, hastening deterioration [75]. FT is considered a “green” procedure that does not require chemicals or a lot of energy. Furthermore, FT cycles occur naturally in a variety of areas around the world without the need for energy [52]. Contrariwise, a significant amount of energy is required in other parts of the world to achieve and maintain the required temperature. Table 7 shows a comparative analysis of literature examples that investigated FT potential as a complementary pretreatment method. As can be observed, the results obtained in this study for two cycles of FT followed by alkaline extraction fit the trends presented in the literature. In addition, the proposed method has the benefit of a significantly reduced freezing time, which directly correlates with a reduction in energy consumption.

#### 3.6.2. Microwave Pretreatments

Unlike conventional ovens that transmit heat by conduction–convection mechanisms, exposure to a microwave field generates heat inside the material, the heat transfer and energy yield being significantly higher. Microwaves do not directly affect the molecular structure, and heat propagation is realized by two mechanisms: ionic conduction and dipole rotation [78]. During microwave pretreatments of lignocellulosic biomass, the structure of the biomass can be swelled and fractured due to microwave-generated oscillations: dipolar rotation and molecular collisions [79]. The presence of water speeds up heat transfer and improves the hydrolysis process. The “thermal” and/or “non-thermal” effects of MW are still under debate [46,80]. The main advantages of MW complementary treatments are: reduction in chemical consumption, energy, and reaction time [26,46,78]. However, there are a few drawbacks related to the unequal distribution of microwave power inside non-homogeneous materials that may generate hot spots (local overheating); the energy absorption depends upon the dielectric properties of the material [78]. Table 8 presents a comparative analysis of MW-assisted wheat straw alkaline extraction. The difference in efficiency between the reported data and the results obtained in this work is because no additional treatment was applied (HA), which indicates that MW can be used as a standalone extraction procedure. It is worth mentioning that the reported yield was obtained only after 10 min of MW.

#### 3.6.3. Ultrasound Pretreatments

Ultrasound exposure can generate both physical and chemical effects. Cavitation phenomena (formation, growth, and implosive bubble collapse) produce huge temperatures and high pressures for extremely short periods [82]. The corresponding shock waves enhance energy and mass transfer. Acoustic cavitation at high frequencies generates highly reactive free radicals, such as hydroxyl, superoxide, or singlet oxygen [83]. The main features of US employment as complementary pretreatment are reducing processing time, lowering the temperature and pressure, and diminishing the amount of chemicals [41]. The main drawbacks of US-assisted pretreatments are related to the sonic field’s uniformity and reduced possibilities of upscaling the procedure, caused by the transducers overheating [41,82]. Nevertheless, some modern ultrasound reactors equipped with multiple transducers can surpass these inconveniences [41,82]. Table 9 shows that US pretreatment alone has a reasonable extraction yield concerning the time used.

#### 3.6.4. SEM Analysis

**Figure 9:**
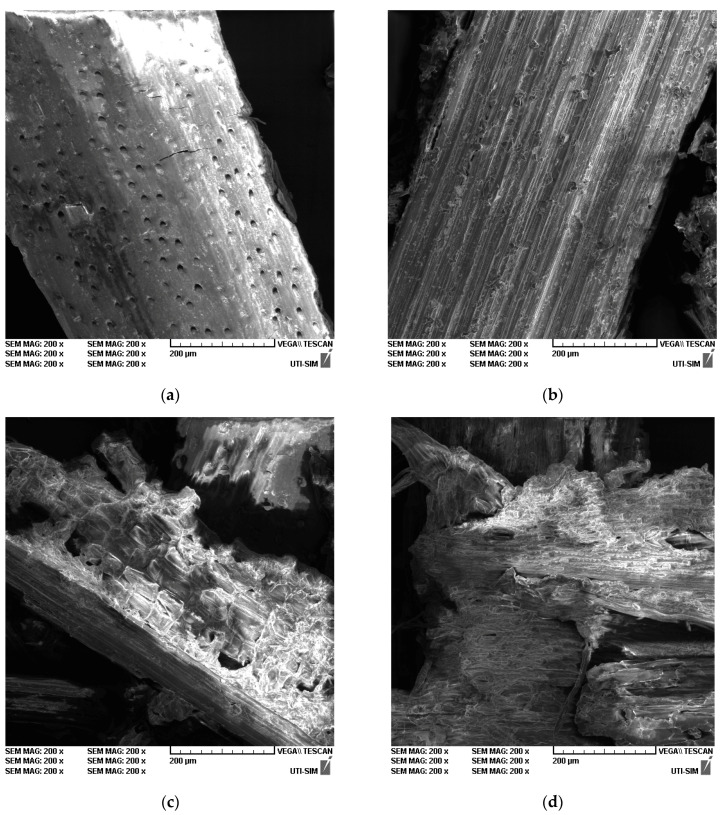
SEM images of wheat straw after various treatments: (**a**) wheat straw without any treatment; (**b**) wheat straw after alkali treatment; (**c**) wheat straw after the freeze–thaw cycle; (**d**) wheat straw after freeze–thaw and alkali treatment.

The samples’ surface morphology, studied at 200 × magnification and at a working distance of about 42 mm, are presented in Figure 9a–d. Figure 9a shows the secondary electrons image of wheat straw without any treatment. The damaging effect of alkali on the straw protective shell, exposing the inner fibers, is revealed in Figure 9b. Figure 9c demonstrates the disruptive effects of ice formation during freezing, as discussed in Section 3.6.1. Finally, the combination of freezing and alkali effects are displayed in Figure 9d, where it can be observed that the fibers and cells are exposed and fractured. Since freezing causes mechanical damage, it sets the stage for alkaline action deep within the material.

## 4. Conclusions

The performance of three complementary pretreatments: freeze–thaw cycles, ultrasound, and microwaves, was investigated in an experimental trial for the hot alkaline extraction of hemicelluloses from wheat straw. During trials, it was discovered that two freeze–thaw cycles are sufficient to achieve satisfactory extraction yields.

The severity factor and alkali concentration were subsequently considered variables in a multi-objective optimization study for two freeze–thaw cycles followed by hot alkaline extraction. The predicted values were experimentally validated: (i) considering an optimal value within the range of process parameters; and (ii) considering an optimal value that extrapolates the process parameters. For the first case, the optimal conditions are represented by CNaOH = 7% and SF = 1.63. The hemicelluloses obtained in these conditions were then analyzed for purity (96.8%); and glucan (3.40%), xylan (85.51%), and arabinan (7.89%) content.

Although microwaves and ultrasounds outperformed the freeze–thaw procedure in terms of extraction yields, technical issues, such as temperature control, energy consumption, simplicity, the possibility of preserving the polymeric structure of the resulting materials, and upscaling perspectives, recommend the combination of classic hot alkaline extraction with freeze–thaw pretreatments for wheat straw processing.

## Figures and Tables

**Table 1:** Independent variables and variation range for hemicelluloses extraction from wheat straw.

Independent Variables	Units	Label	Range		Symbol
			From	To	
Severity factor	-	X1	1	3	SF
NaOH concentration	% wt.	X2	3	7	CNaOH

**Table 2:** Chemical composition of wheat straw varieties as raw materials in the study.

Variety	Cellulose % (Std)	Xylan % (Std)	HC % (Std)	AIL % (Std)	ASL % (Std)	AE % (Std)	HWT % (Std)	Ash % (Std)
Otilia	39.64 (0.98)	21.62 (1.14)	24.97 (0.38)	18.67 (1.12)	1.95 (0.12)	2.17 (0.03)	15.47 (0.02)	5.57 (0.06)
Sorial	39.73 (0.45)	18.79 (1.88)	26.01 (0.24)	15.8 (0.48)	2.45 (0.11)	4.09 (0.04)	18.08 (0.41)	6.44 (0.17)
Izvor	40.9 (0.59)	20.56 (1.37)	27.27 (0.31)	20.6 (0.11)	1.83 (0.16)	2.49 (0.05)	13.6 (0.5)	4.88 (0.14)
Mixture of WS (1:1:1)	40.1 (1.12)	20.23 (1.1)	26.2 (1.78)	16.36 (1.1)	1.48 (0.22)	2.91 (0.15)	15.66 (0.18)	5.61 (0.35)

Results presented as mean of triplicates; std represents the standard deviation.

**Table 3:** The effect of freeze–thawing cycles on the subsequent yields obtained in alkaline extraction.

Sample Treatment	TY (%)	XY (%)	HCY (%)	YAIL (%)	YASL (%)
HA 40-90	42.21	24.16	34.03	25.9	27.5
HA 40-100	44.2	36.1	35.8	32.08	35.1
FT2	7.50	3.72	3.08	2.68	0.89
US10-30	31.33	4.38	9.28	19.16	11.22
US20-45	34.40	21.67	21.96	24.85	18.9
MW 10-100	52.18	31.51	31.39	29.40	19.91
FT 1 HA 40-90	45.2	58.41	60.02	23.69	37.81
FT 2 HA 40-90	47.3	61.75	63.43	25.66	39.8
FT 3 HA 40-90	46.6	58.19	56.51	23.66	38.2
FT 4 HA 40-90	45.1	56.68	54.80	24.23	38.9

**Table 4:** Experimental results and conditions of hot alkaline extraction of wheat straw.

Exp.	SF	CNaOH (%)	TY (%)	XY (%)	HCY (%)	YAIL (%)	YASL (%)
1	1	3	34.86	15.24	19.24	19.67	28.42
2	2	3	49.79	34.75	34.87	13.65	24.66
3	3	3	49.58	50.15	48.28	56.05	28.77
4	1	7	41.60	52.57	50.72	27.55	32.19
5	2	7	56.01	63.84	61.70	26.24	45.89
6	3	7	54.21	70.84	70.98	75.46	48.56
7	1	5	40.22	49.65	49.06	38.59	31.44
8	3	5	51.43	55.60	57.23	60.21	44.86
9	2	5	49.76	48.44	46.98	20.97	36.30
10	2	5	50.55	54.32	52.23	21.03	34.93
11	2	5	49.73	49.68	42.46	20.77	39.04
12	2	5	50.36	45.11	47.10	25.94	35.62
113	2	5	50.22	49.18	48.45	19.67	36.30

**Table 5:** The optimized conditions of wheat straw hemicellulose extraction and obtained experimental results.

		TY (%)	XY (%)	HCY (%)	YAIL (%)	YASL (%)
O1	Predicted model parameters	CNaOH = 7%; SF = 1.63				
	Predicted results	51.56	59.97	57.07	21.58	39.60
	Experimental validation	49.94	62.56	61.25	20.72	38.63
O2	Predicted model parameters	CNaOH = 9%; SF = 1.44				
	Predicted result	55.9	67.71	63.69	24.10	23.62
	Experimental validation	50.34	52.20	52.05	19.64	19.70

**Table 6:** Main chemical components of hemicelluloses preparations isolated from wheat straw.

Sample	Glucan (%)	Xylan (%)	Arabinan (%)	Purity (%)
HC_C (control)	3.61	77.50	13.14	94.25
HC_O1	3.40	85.51	7.89	96.80
HC_O2	2.61	83.92	10.02	96.55

**Table 7:** Comparison of freeze–thaw enhancement of agro-waste biomass pretreatments.

Biomass Type	Extraction Procedure	No. of FT Cycles	Temperature (°C)	Freezing Time (h)	Reported Extraction Yields ^*^	Reference No., Year
wheat straw	enzymatic hydrolysis	1	−10	12, 24, 48, 96	57.06% cellulose, 70.66% hemicelluloses	[75], 2022
			−20			
			−40			
			−80			
wheat straw	enzymatic hydrolysis	1	−20	48	75.95% cellulose and hemicelluloses	[52], 2012
wheat straw	enzymatic hydrolysis	2	−20	12	67% cellulose and hemicelluloses	[10], 2013
bamboo chips	alkaline extraction	1	−30	12	64.71% hemicelluloses	[76], 2021
bamboo chips	alkaline extraction	1	−20	12	73.26% hemicelluloses	[77], 2022
			−30			
			−40			
			−50			
			−60			
			−70			
wheat straw	FT standalone	2	−22	1	3.8% hemicelluloses	this work, 2023
	alkaline extraction				34.03% hemicelluloses	
	FT + alkaline extraction				63.43% hemicelluloses	

* Highest reported values.

**Table 8:** Comparison of MW-assisted wheat straw alkaline extraction.

Alkali Concentration Range (%wt)	Temperature Range (°C)	Time Range (Minutes)	Reported Yields *	Reference No., Year
0.5–2.5	120–200	5–25	69.48% of lignin solubilized	[79], 2019
1–5	60–140	5–80	90.66% purity of cellulose	[46], 2021
2–5	60–140	10–60	80% of hemicelluloses 90% of lignin	[81], 2012
5	100	10	31.39% hemicelluloses	this work, 2023

* Highest reported values.

**Table 9:** Comparison of US-assisted wheat straw pretreatments.

Extraction Procedure	US Frequency (kHz)	Power (W)	Time, (Minutes)	Solvent	Reported Extraction Yields *	Reference No., Year
enzymatic hydrolysis	40	200	60, 120	water, diluted H_2_SO_4_, diluted NaOH	59.2% lignin removal	[41], 2022
	25	2000				
		3000				
organosolv	20	100	5, 10, 15, 20, 25, 30, 35	solution NaOH/methanol/water	78.5% lignin removal	[84], 2002
deep eutectic solvents	40	200	60, 120	acetic acid/glycerol/ choline chloride	27% delignification	[85], 2018
				γ-valerolactone/ water		
ammonia	20	180, 270, 450, 540, 650	15, 30, 45, 60, 90	water	92% saccharification	[19], 2017
alkaline extraction	20	100	5, 10, 15, 20, 25, 30, 35	KOH	solubilization 91.6% hemicelluloses, 91.4% lignin	[86], 2005
standalone ultrasonic treatment	40	50	10	NaOH	9.28% hemicelluloses	this work, 2023
			20		21.96% hemicelluloses	

* Highest reported values.

## Data Availability

The data supporting the reported results are presented in the manuscript.

## Supplementary Materials

The following supporting information can be downloaded at: https://www.mdpi.com/article/10.3390/polym15041038/s1, Figure S1: Complete FTIR spectra of separated hemicelluloses; Table S1: ANOVA analysis results for the response model of the total extraction yield TY (%);Table S2 ANOVA analysis results for the response model of the xylan extraction yield XY (%); Table S3 ANOVA analysis results for the response model of the total hemicelluloses extraction yield HCY (%); Table S4 ANOVA analysis results for the response model of the acid insoluble lignin removal yield YAIL (%); Table S5 ANOVA analysis results for the response model of the acid soluble lignin removal yield YASL (%).
