# Supplementary material for: Optimization of Alkaline Extraction of Xylan-Based Hemicelluloses from Wheat Straws: Effects of Microwave, Ultrasound, and Freeze–Thaw Cycles
Source: Polymers (Basel). 2023 Feb 19;15(4):1038. doi: 10.3390/polym15041038 (PMC9963123; doi:10.3390/polym15041038)
Supplement: Supplementary file 1 [file polymers-15-01038-s001.zip › polymers-2218224-supplementary.pdf]

Supplementary material

Optimization of alkaline extraction of xylan-based hemicelluloses from wheat straws: effects of microwave, ultrasound and freeze-thaw cycles

Adrian Cătălin Puițel [1], Gabriel Dan Suditu [1], Elena Niculina Drăgoi [1], Maricel Danu [1,2], Gabriela-Liliana Ailiesei [2], Cătălin Dumitrel Balan [1], Daniela-Lucia Chicet [3] and Mircea Teodor Nechita [1,*]

[1] "Cristofor Simionescu" Faculty of Chemical Engineering and Environmental Protection, "Gheorghe Asachi" Technical University, Bd. Prof. Dimitrie Mangeron, No. 73, 700050 Iaşi, Romania
[2] "Petru Poni" Institute of Macromolecular Chemistry, 41A Grigore Ghica Voda Alley, 700487 Iași, Romania
[3] Faculty of Materials Science and Engineering, "Gheorghe Asachi" Technical University, Bd. Prof. Dimitrie Mangeron, No. 41, 700050 Iaşi, Romania
* Correspondence: mircea-teodor.nechita@academic.tuiasi.ro

The complete FTIR spectra for the hemicelluloses materials obtained in the control case (HC_C), optimized conditions considering objective O1 (HC_O1) and the optimized conditions determined for objective O2 (HC_O2) is presented in Figure S1.

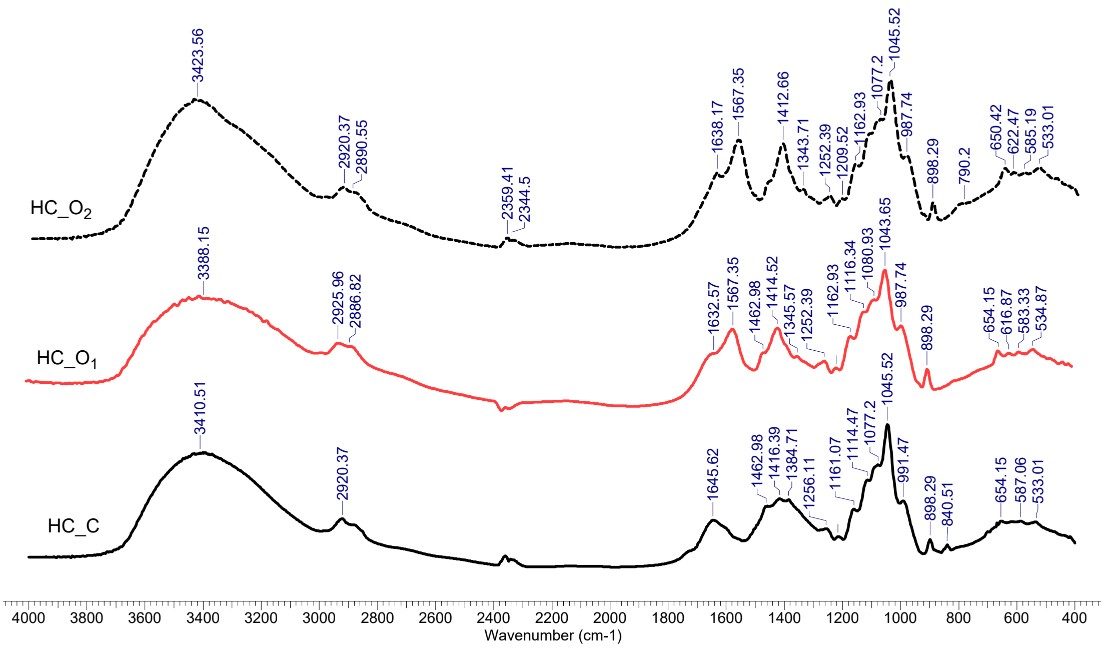

Figure S1. Complete FTIR spectra of separated hemicelluloses

Tables S1-S5 present the ANOVA analysis for the models determined for the 5 considered outputs of the process. In all cases, the models have a p-value<0.05, indicating that the determined models are statistically significant.

Table S1. ANOVA analysis results for the response model of the total extraction yield TY (%)

Response TY (%)					
Source	Sum of Squares	df	Mean Square	F Value	p-value Prob > F
Model	404.73	5	80.95	50.278	< 0.0001
SF	247.56	1	247.56	153.75	< 0.0001
CNaOH (%)	51.57	1	51.57	32.03	0.000767
SF* CNaOH (%)	1.11	1	1.11	0.69	0.433
SF^2	99.68	1	99.68	61.91	0.000101
CNaOH $(\%)^2$	3.15	1	3.15	1.95	0.205
Residual	11.27	7	1.61		
Lack of Fit	10.74	3	3.58	26.86	0.00413
Pure Error	0.53	4	0.13		
Cor Total	415.99	12			
			R-Squared: 0.973<br>Adj R-Squared: 0.954		

Table S2 ANOVA analysis results for the response model of the xylan extraction yield XY (%)

Response XY (%)					
Source	Sum of Squares	df	Mean Square	F Value	p-value Prob > F
Model	2064.16	3	688.05	52.81	< 0.0001
SF	729.94	1	729.94	56.03	< 0.0001
CNaOH (%)	1264.91	1	1264.91	97.09	< 0.0001
SF* CNaOH (%)	69.30	1	69.30	5.32	0.0465
Residual	117.25	9	13.028		
Lack of Fit	73.60	5	14.72	1.35	0.397
Pure Error	43.65	4	10.91		
Cor Total	2181.41	12			
			R-Squared: 0.95<br>Adj R-Squared: 0.928		

Table S3 ANOVA analysis results for the response model of the total hemicelluloses extraction yield HCY (%)

Response HCY (%)					
Source	Sum of Squares	df	Mean Square	F Value	p-value Prob > F
Model	1763.01	3	587.67	47.54	< 0.0001
SF	649.81	1	649.81	52.57	< 0.0001
CNaOH (%)	1093.89	1	1093.891	88.50	< 0.0001
SF* CNaOH (%)	19.29	1	19.29	1.56	0.243
Residual	111.24	9	12.36		

Lack of Fit	62.13	5	12.43	1.012	0.510
Pure Error	49.12	4	12.28		
Cor Total	1874.24	12			
			R-Squared: 0.94<br>Adj R-Squared: 0.92		

Table S4 ANOVA analysis results for the response model of the acid insoluble lignin removal yield YAIL (%)

Response YAIL (%)					
Source	Sum of Squares	df	Mean Square	F Value	p-value<br>Prob > F
Model	4291.91	5	858.38	84.15	< 0.0001
SF	2192.90	1	2192.90	214.97	< 0.0001
CNaOH (%)	265.19	1	265.19	25.99	0.00140
SF* CNaOH (%)	33.29	1	33.29	3.26	0.114
SF^2	1589.96	1	1589.96	155.87	< 0.0001
$CNaOH\ (\%)^2$	3.15	1	3.15	0.31	0.596
Residual	71.41	7	10.20		
Lack of Fit	47.71	3	15.90	2.69	0.182
Pure Error	23.69	4	5.92		
Cor Total	4363.31	12			
			R-Squared: 0.984<br>Adj R-Squared: 0.972		

Table S5 ANOVA analysis results for the response model of the acid soluble lignin removal yield YASL (%)

Response YASL (%)					
Source	Sum of Squares	df	Mean Square	F Value	p-value<br>Prob > F
Model	550	3	183	24.1	0.000123
SF	151	1	151	19.9	0.00157
CNaOH (%)	334	1	334	44	< 0.0001
AB	64.2	1	64.2	8.44	0.0174
Residual	68.5	9	7.61		
Lack of Fit	58.7	5	11.7	4.81	0.0766
Pure Error	9.76	4	2.44		
Cor Total	618	12			
			R-Squared: 0.889<br>Adj R-Squared: 0.852		
